# Supplementary material for: The role of the locus coeruleus in eye movements during perceptual decision making
Source: bioRxiv. 2026 Mar 3:2026.03.01.708911. Preprint. [Version 1] doi: 10.64898/2026.03.01.708911 (PMC12991126; doi:10.64898/2026.03.01.708911)
Supplement: Supplement 1 [file NIHPP2026.03.01.708911v1-supplement-1.pdf]

bioRxiv preprint doi: https://doi.org/10.64898/2026.03.01.708911; this version posted March 3, 2026. The copyright holder for this preprint (which was not certified by peer review) is the author/funder, who has granted bioRxiv a license to display the preprint in perpetuity. It is made available under aCC-BY 4.0 International license.

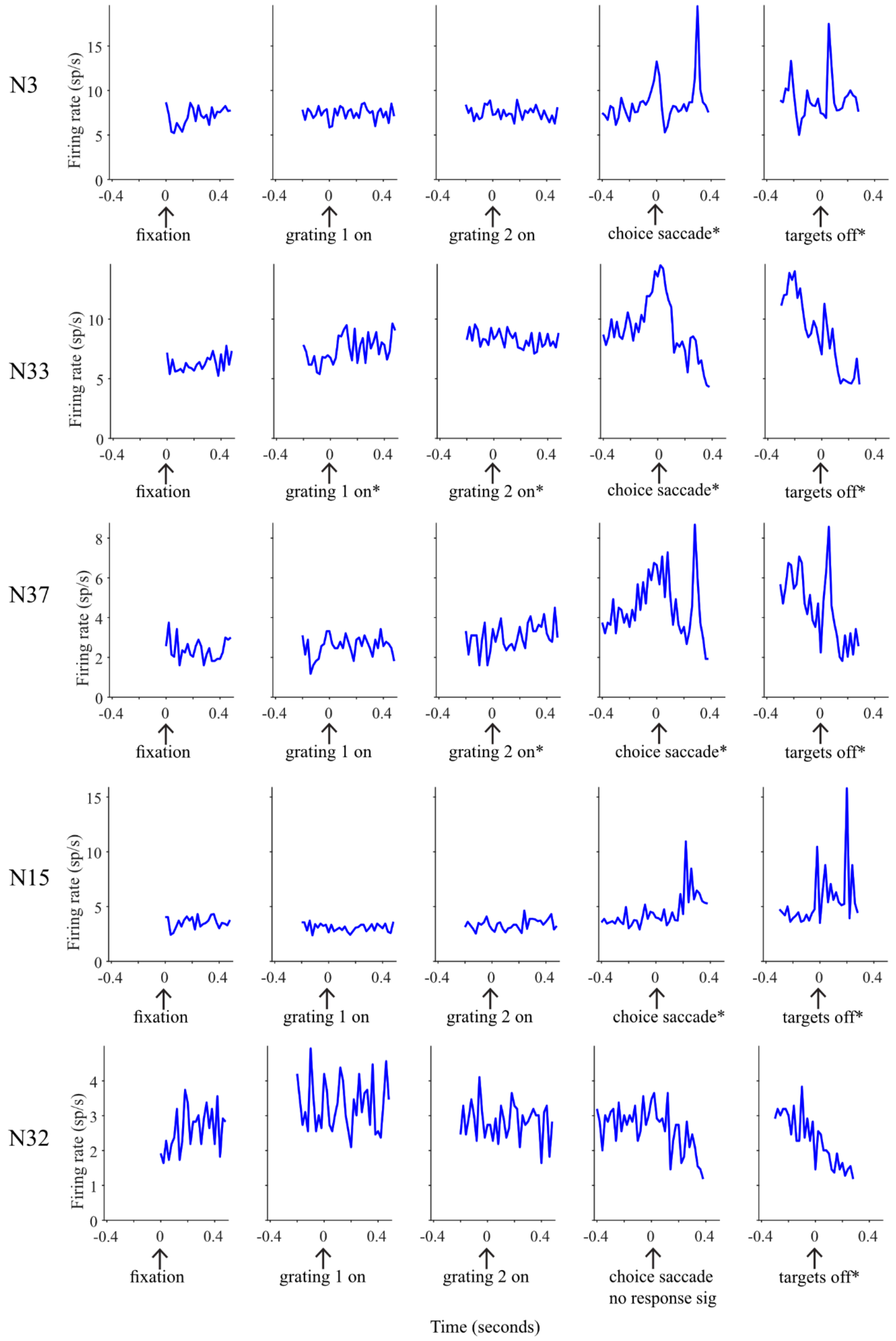

Figure 3 Supplement 1. Example PETHs are shown for 5 neurons (rows) aligned to fixation (column 1), first stimulus grating onset (column 2), second stimulus grating onset (column 3), choice saccade initiation (column 4), and target offset (column 5). Neurons that had a significant change in response relative to baseline have an asterisk next to the plot label text for that condition.

bioRxiv preprint doi: https://doi.org/10.64898/2026.03.01.708911; this version posted March 3, 2026. The copyright holder for this preprint (which was not certified by peer review) is the author/funder, who has granted bioRxiv a license to display the preprint in perpetuity. It is made available under aCC-BY 4.0 International license.

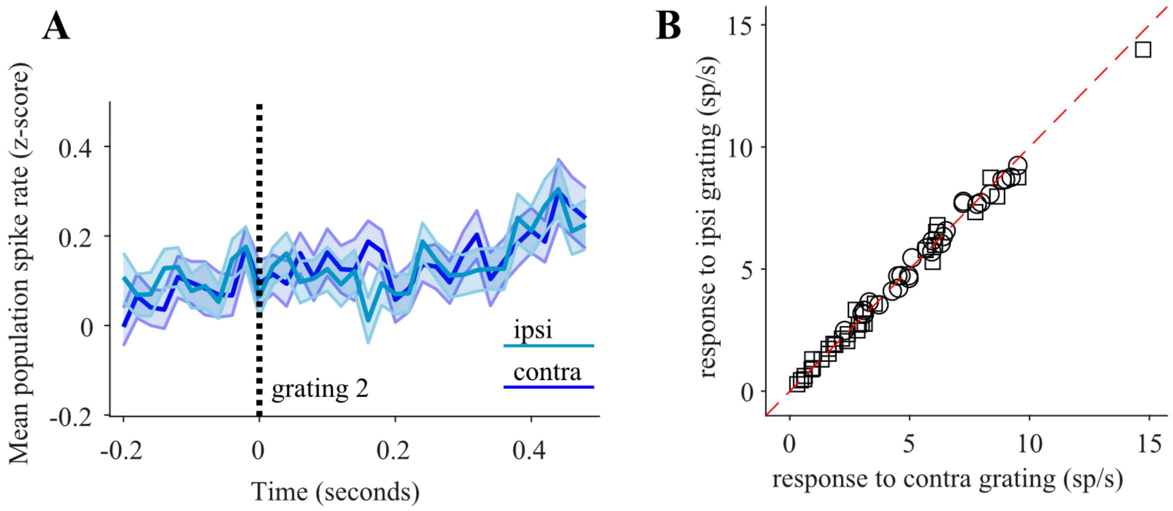

Figure 3 Supplement 2. Task-related LC phasic activation during the test grating interval. (A) We did not observe any transient visual response to the appearance of the second grating, and (B) spike rates during the second grating interval showed no evidence of spatial selectivity based on whether the grating was presented ipsilateral or contralateral to the recorded LC neuron.

bioRxiv preprint doi: https://doi.org/10.64898/2026.03.01.708911; this version posted March 3, 2026. The copyright holder for this preprint (which was not certified by peer review) is the author/funder, who has granted bioRxiv a license to display the preprint in perpetuity. It is made available under aCC-BY 4.0 International license.

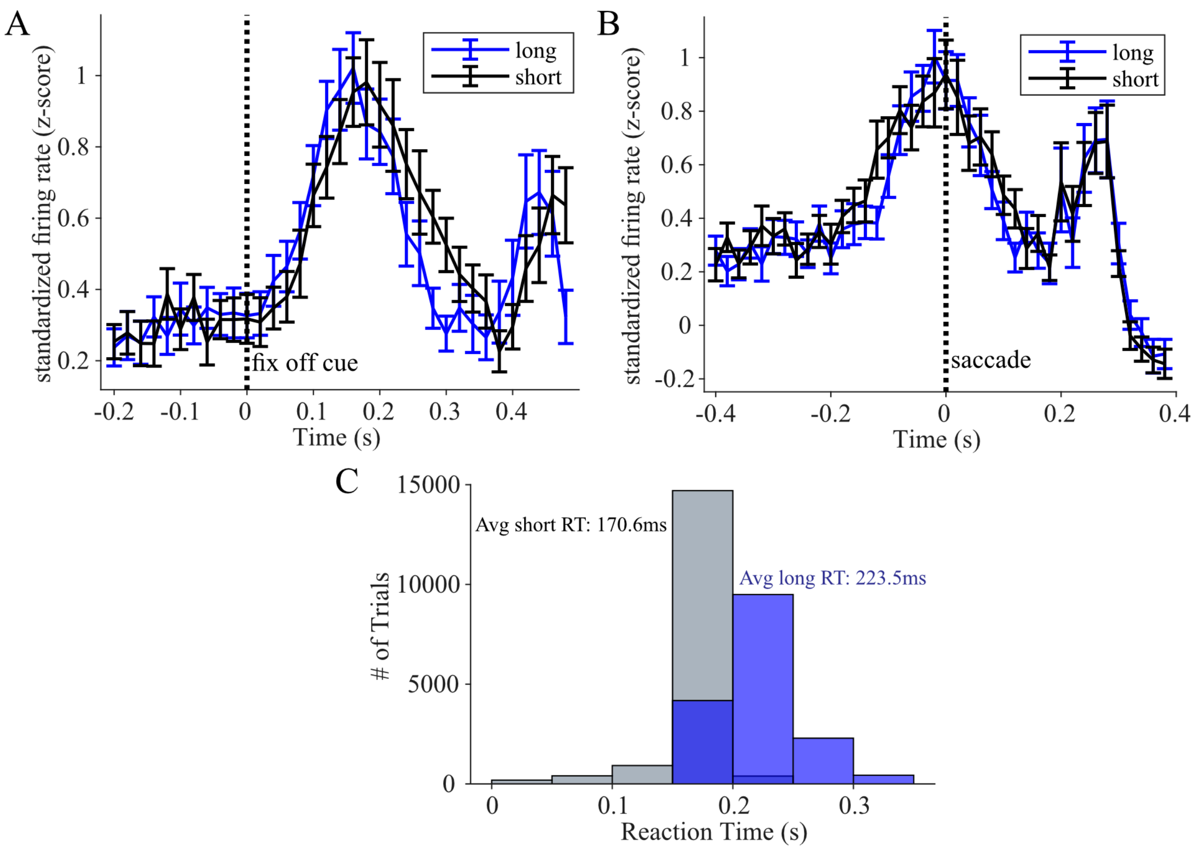

Figure 4 Supplement 1. Comparing LC phasic activation between long and short behavioral response times. (A) Average PETHs compiled across choice target-onset aligned LC responses on long (blue) vs short (black) reaction time (RT) trials (lines and error bars represent mean ± SEM across PETHs of all individual neurons across both monkeys). Target-onset aligned LC firing rates were not significantly different between trials of short vs long RTs (paired t-test, $p = 0.89$). (B) same as (A) but for saccade-aligned LC responses; no significant difference between long and short RT trials (paired t-test, $p = 0.5$).(C) Histograms depict averages and distributions of behavioral response times in the short (gray) and long (blue) RT groups across sessions and monkeys. Long and short RT groups were determined by the median RT in each session.

bioRxiv preprint doi: https://doi.org/10.64898/2026.03.01.708911; this version posted March 3, 2026. The copyright holder for this preprint (which was not certified by peer review) is the author/funder, who has granted bioRxiv a license to display the preprint in perpetuity. It is made available under aCC-BY 4.0 International license.

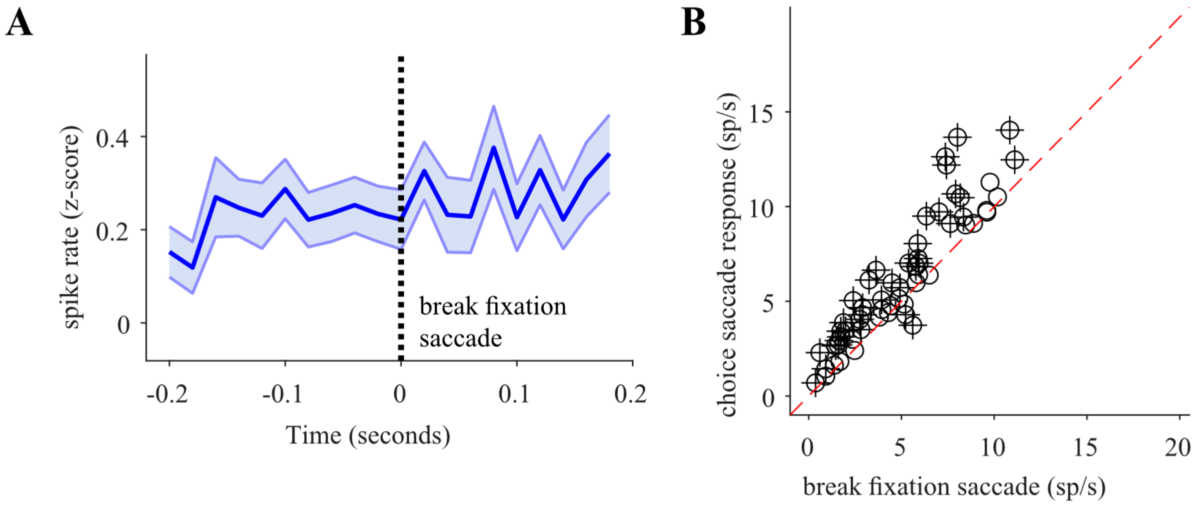

Figure 8 Supplement 1. Relationship of LC activity to non-choice saccades. In addition to task-relevant behavior, in some cases the animals made a saccade away from fixation either to one of the gratings or elsewhere on the screen. These saccades were deemed task-irrelevant, were not rewarded and the trials were halted. (A) Average PETH across all standardized LC responses aligned on onset of task-irrelevant saccades that broke the animal's fixation. (B) Scatter plot depicting the firing rate responses of 59 individual LC neurons aligned on the onset of the task-irrelevant break fixation saccades as compared to task-related choice saccades made during the choice period (y-axis). The majority of recorded neurons showed a significantly greater activation in response to task-related saccades (+ symbols indicate significant differences between the two responses, paired t-test, $p < 0.05$).
